# Participant evaluation of an education module on interprofessional collaboration for students in healthcare studies

**DOI:** 10.1186/s12909-015-0477-0

**Published:** 2015-10-27

**Authors:** Giannoula Tsakitzidis, Olaf Timmermans, Nadine Callewaert, Steven Truijen, Herman Meulemans, Paul Van Royen

**Affiliations:** 1 Department of Primary and Interdisciplinary Care, Faculty of Medicine and Health Sciences, University of Antwerp, Antwerp, Belgium; 2 Department of Nursing and Midwifery Sciences, Faculty of Medicine and Health Sciences, Centre for Research and Innovation in Care, University of Antwerp, Antwerp, Belgium; 3 HZ University of Applied Sciences, Vlissingen, The Netherlands; 4 Department of Health Sciences, Artesis-Plantijn University College of Antwerp, Antwerp, Belgium; 5 Department of Rehabilitation Sciences and Physiotherapy, Faculty of Medicine and Health Sciences, University of Antwerp, Antwerp, Belgium; 6 Department of Sociology and Research Centre for Longitudinal and Life Course Studies, University of Antwerp, Antwerp, Belgium; 7 Centre for Health Systems Research and Development, University of the Free State, Bloemfontein, South Africa

**Keywords:** Interprofessional, Collaborate, Education, Healthcare

## Abstract

**Background:**

Interprofessional collaboration is considered a key-factor to deliver the highest quality of care. Interprofessional collaboration (IPC) assumes a model of working together, in particular with awareness of the process of interprofessional collaboration, to develop an integrated and cohesive answer to the needs of the client/family/population. Educational modules are developed in response to a perceived need to improve interprofessional collaboration for the benefit of patientcare. Up until 2005 no explicit module on interprofessional collaboration existed in the education programs of the Antwerp University Association (AUHA). During a decade the ‘Interprofessional Collaboration In Healthcare (IPCIHC) – module’ is organised and evaluated by its participants.

**Methods:**

One group, post-test design was used to gather data from the participating students using a structured questionnaire. Data was collected between March 2005 and March 2014 from participating final year students in healthcare educational programs.

**Results:**

3568 (84 % overall response) students evaluated the IPCIHC module from 2005 up to 2014. Over 80 % of the participants were convinced the IPCIHC increased their knowledge and changed their understanding that it will impact their future professional relationships, and felt a greater understanding about problem-solving in healthcare teams. Even though the results indicate that the goals of the IPCIHC module were achieved, less than 60 % of the participants experienced a change in attitude towards other professional groups.

**Conclusions:**

Despite the positive outcomes from the participants, the challenge still remains to keep on educating future healthcare providers in interprofessional collaboration in order to achieve an increase in interprofessional behaviour towards other professional groups. Research is needed to investigate the effectiveness of undergraduate programs on the quality and safety of patientcare in practice.

## Background

Flexner wrote in his report [1] “An education in medicine involves both learning and learning how; the student cannot effectively know, unless he knows how”. One hundred years later it seems that with transformative learning healthcare workers develop their leadership and learn to collaborate interprofessionally in teams so they can contribute to changes in society and healthcare [2]. Healthcare professionals in Belgium continue to be educated in silo-structured mono-disciplinary educational systems, wherein interprofessional collaboration is taught through clinical practice through learning by doing [3]. Seemingly interprofessional teamwork evolves from trial and error learning [4] and so interprofessional collaboration (IPC) has to be actively taught [5, 6]. Overall IPC assumes a model of working together [7], in particular with awareness of the process by which healthcare professionals develop an integrated and cohesive answer to the needs of the client/family/population [8] with a common vision and purposeful approach and with shared responsibility [9].

The rapidly changing context in healthcare, with increasingly more chronic and multimorbid pathologies, resulting in complex care situations and with more professionals involved, sets clear demands towards IPC [10]. Educational modules on interprofessional collaboration are developed in response to a perceived need to improve interprofessional collaboration for the benefit of patientcare [11]. However, the emphasis on interpersonal skills as a key feature of successful interprofessional working [12] logically should imply that students also have opportunities to interact face-to-face with other students and professionals [13]. Additionally it seems that, unless senior staff in both environments fully support interprofessional initiatives, it is extremely difficult for teaching staff to ensure that students have suitable opportunities to learn and work interprofessionally [13]. Up until 2005 no explicit course of interprofessional learning was organised in the education programs of the Antwerp University Association (AUHA) [3]. To address this problem, we developed the ‘Interprofessional Collaboration In Healthcare (IPCIHC) – module’. This interprofessional education module for pre-licensure students is now organised in the education programs of the Antwerp University Association (AUHA) [14]. We have implemented the IPCIHC module annually for 10 years. As in Hunters’ study [15], the goal in our project was to effectively translate interprofessional collaboration in healthcare into an educational format where pre–licensure students could learn with, from and about each other [16]. During the following ten consecutive years, annual evaluations were used from students, teachers, faculty and participating institutions, to continually refine content, improve process and integrate the most current teaching methods, research on IPC and interprofessionalism. The IPC committee members representing all ten faculties and departments of the participating institutes, provided ongoing input into the yearly modifications in working with this challenge for curriculum design as well as for challenging the organisation in regards to the ‘face - to - face’ program. This paper describes the participant evaluation of the interprofessional module on items such as knowledge, attitude and future relationships with other professionals in healthcare. In addition, the study reports on the development of this interprofessional learning module.

## Methods

### Development of the program

The starting point of the development of the interprofessional module was the setup of an interprofessional steering team with representatives from the participating education programs. All the education programs for healthcare of the Antwerp University Association (AUHA) received an invitation for participating in developing an interprofessional education module. The aim of the project was to develop an integrated interprofessional module in the existing participating educational programs in the region of Antwerp city. Additionally it had to conform to the existing learning goals for every participating education program. The interprofessional module had to be organised based on competence according to the Bologna Declaration of 19 June 1999 so that widespread student mobility could be promoted. Finally the importance of assessment had to be respected and appropriate instruments to assess the competence ‘interprofessional collaborator’ had to be used. The first ‘Interprofessional Collaboration In Healthcare (IPCIHC) – module’ was organised in March 2005.

#### Structure of the IPCIHC-module: learning and assessment methods used

Even though different terms are often used in literature to define a model of working together, [7] particularly with regards to the process [8], the term ‘interprofessional’ was consistently used in the IPCIHC-module to create a common language [17]. To help participants in developing a common vision in the IPCIHC-module, the model of team effectiveness of Fry [18] was used. A team should always begin with a team level goal. After the goal is defined, the roles and responsibilities will become clearer. As individuals work together, they will see that goals and responsibilities are often not sufficiently clear. Consequently, team members will need to redefine them. That redefinition enables them to adjust and readjust team processes, such as decision making, conflict resolution and work flow. When doing all that, they will be developing the interpersonal relationships needed to relate to other team members and the team leader [19]. In this module the Flexner report is also taken into account to learn and to learn how to collaborate, by using Miller’s pyramid of clinical competence [20]. The definition of CAIPE [16], used in this module as a central concept, also helped to make choices about didactical and methodological teaching tools when developing the IPCIHC-module.

The definition of the Centre For The Advancement Of Interprofessional Education (CAIPE) was used: “Interprofessional education occurs when two or more professions learn with, from and about each other to improve collaboration and the quality of care” [16].

**Fig. 1:**
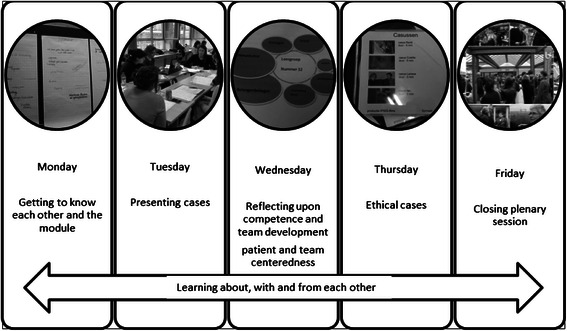
Structure and main components of the one week IPCIHC model

The recurring theme in the learning process in the IPCIHC-module was competency oriented, where students were assessed based on Miller’s learning pyramid [20]. The IPCIHC-module was presented in one week (see Fig. 1) for all final year students of the participating institutions: physicians, physiotherapists, occupational therapists, nurses, midwives, dieticians, speech therapists, social workers and bachelors in psychology. Proper assessment tools to evaluate the learning goals were portfolio, self – and peer assessment and group evaluation. The curriculum contained colleges (3 plenary sessions with lecturers), workshops coached by one teacher and practical sessions for case studies and creation of care plans. We used basic principles of interprofessional learning in terms of learning-teaching issues, such as collaborative learning, egalitarian learning, group-oriented learning: shared responsibility, learning through experience, reflective learning and applied learning [6, 17, 21, 22].

#### Content and process

##### Plenary sessions

In order to become familiar with the goals and definition on interprofessional collaboration the IPCIHC-module started with a plenary introductive session on the Monday morning. In the entire group (between 270 and 600 students, depending on the academic year) some brief lectures were given, alternating with videos, about the foundation and building bricks of interprofessional collaboration such as ‘what is interprofessional collaboration’ and ‘explaining the link between education and practice’. To achieve the objectives, the following topics were presented during plenary sessions: leadership and teambuilding, an inventory and analysis of collaboration in healthcare. In total there were three plenary sessions; one on the first day as introduction, the second on the third day with focus on patient and team centeredness and the closing session with a multidisciplinary panel. The multidisciplinary panel was a representation of patients, policy and healthcare providers.

##### Small group sessions

From the first day onward (after the plenary session), students were allocated into an interprofessional group of maximum 14 students in which they remained throughout the whole module. The learning goal on the first day started with getting acquainted interprofessionally. So participants in the learning group delved into their own profession and what they knew about the other professions. On the second day, allocated in interprofessional groups again, they had to create a care plan for a case chosen from real clinical situations (anonymised) taken from their mono-disciplinary program. In order to create the care plan the International Classification of Functioning, Disability and Health was used. This is known more commonly as ICF and is a classification of health and health-related domains [23]. On the third day, in the afternoon, focusing on patient-centeredness and thinking and acting ethically, they had to reflect upon their own development in the seven roles of the competence ‘collaborator in healthcare’, as well as reflect upon the group process and development during the three days up until then. On the fourth day students had to discuss ethical cases presented by the teacher on video. Finally at the end of the fourth day, the group had to prepare their presentation for the last day of the module and fill in the self- and peer assessments. On the final day they presented their results and findings during the plenary session and discussed or directed questions to a multidisciplinary panel.

##### Assessment and evaluation of the students

All results and reflections had to be filed in a portfolio for evaluation. The students’ final score on their competence ‘interprofessional collaborator in healthcare’ was based for 50 % on portfolio and 50 % on ‘collaborative behaviour’ during the IPCIHC-module. Students’ portfolio was evaluated by tutors from their own discipline and the score for ‘collaborative behaviour’ was the result of group evaluation, peer and self-assessment in the interprofessional team.

##### The competences and learning goals

**Fig. 2:**
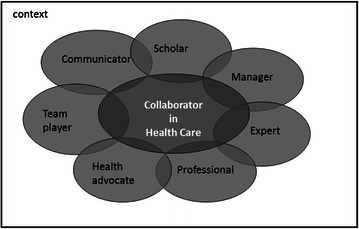
The competence ‘Collaborator in Healthcare’ described in seven roles

Through literary research and discussions with experts, information was gathered and discussed within the steering committee. The representatives of the participating educational programs considered the competences that should be developed to become an effective collaborator in healthcare. The goal of the IPCIHC-module was to prepare all participating health professional students for deliberately working together with the common goal of building a safer and better patient-centred and community/population-oriented healthcare system [24]. As in the team effectiveness model of Fry [18], all student-teams in the IPCIHC-module had to appoint the goal, the roles every member of the team played and the procedures for every assignment during this educational program. The competence for ‘collaborator in healthcare’ was finally described as seven roles based upon the CanMeds roles 2005 [25]. The fundamental difference with the original and medical competence description of the CanMeds roles is that ‘collaborator in healthcare’ is the central competence (see Fig. 2).

For every role the core competencies were described and also used as basis for self- and peer assessment on the competence as ‘collaborator in healthcare’.

The basic principle for interprofessional collaboration in this module was understood as a bio-psycho-social model with patient-centeredness [26] and shared decision-making; so looking at patients as persons who have a problem and who present themselves to a certain health worker with a request for help. The patients tell their story to the healthcare professional and together they search for the best available and relevant treatment thus enabling a team to start developing a care plan. The problem in the patient/client context is the starting point, not as frequently in practice: available discipline tests to see if they can be meaningful in the process of rehabilitation [27].

### Evaluation of the module

Annual evaluations from the students, teachers, departments and faculty were iteratively used to continually refine content, improve process and to integrate the most current interprofessionalism. No structural gathering of those data was used. For this study we therefore focused on the participant evaluation, which was performed annually.

### Study design

A singular group post-test design was used to gather data from the participants using a structured questionnaire. Data was collected between March 2005 and March 2014. On a standardised moment, all participants were offered the questionnaire. No approval of an ethics committee was required for this study according to the Belgian Law of 7 May 2004 concerning Experiments on the Human Person. Therefore the study does not in any way constitute or involve a ‘test carried out on the human person’ (within the meaning of Article 2, 7° of this Law), but only concerns an assessment of an educational module. All participating students were informed about the questionnaire and the fact that the data would be processed anonymously.

### Sample

From all the participating education programs for healthcare of the Antwerp University Association (AUHA) all final year students between 2005 and 2014 were included. The students represented the faculty of medicine and health sciences (University Antwerp), the departments health and social care of the Artesis University College Antwerp, the departments health and social care of the University College Karel-de-Grote Antwerp, the department of dieticians and nutrition of the University College Plantijn and the department of psychology and speech therapy of the University College Lessius.

### Data collection-instrument and analysis

To evaluate whether the learning goals for this interprofessional education module such as knowledge, attitude and future relationships with other professionals in healthcare, were achieved the interprofessional module was progressively evaluated with the participating students. Similar to the evaluation strategy used in Parsell et al. (1998) [28] the participants evaluated the course by a written questionnaire. Seven closed questions were translated from English to Dutch and back to English. For question five and seven the term ‘interprofessional’ was used instead of ‘multiprofessional‘. Also for question six ‘NHS’ (National health service) was replaced by ‘Healthcare’. The seven closed questions were:

1. Has the course increased your knowledge of the roles and duties of other professional groups?
2. Has the course changed your understanding of how other professional groups work?
3. Has the course changed your attitude towards other professional groups?
4. Do you feel that a course in interprofessional learning will have any effect on your future relationships with other professional groups?
5. Should interprofessional learning be included in your undergraduate course?
6. Do you feel you have a greater understanding about problem solving in teams in the healthcare?
7. Do you think a course in interprofessional learning will enable you to work more effectively as a member of a healthcare team?

All questions could be answered simply with ‘yes’ or ‘no’. Missing answers were scored severely as ‘no’. To increase the response rate, tutors were asked to gather the questionnaires on the last day of the module. Questionnaires were filled in anonymously.

Descriptive statistics were used to summarize and describe the data gathered by the questionnaire.

## Results

**Fig. 3:**
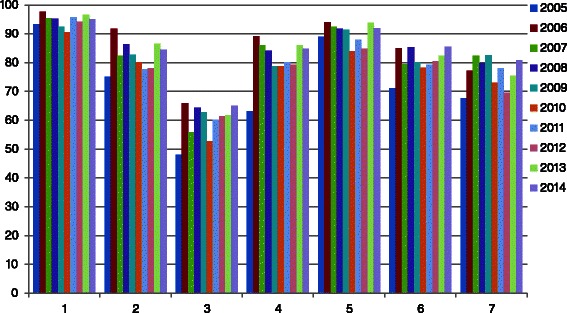
Percentage of ‘yes’ answers per question per year on the seven closed questions (Total number of participants = 3568)

Since 2005, over 4000 students have attended this module. All participant evaluations and comments from 2005 up to 2014 were gathered (see Fig. 3). The evaluation was anonymous and in total 3568 of 4232 (84 % overall response) students evaluated the module.

Overall 90 % of all participants indicated that the IPCIHC-module increased their knowledge about the roles and duties of other professional groups. 80 % was convinced the IPCIHC-module changed their understanding on how other professional groups work. Less than 60 % of the participants experienced a change in attitude towards other professional groups. The participants commented that they already had a positive attitude before the IPCIHC-module. The percentage of positive scores for this question increased between 2005 and 2014. On the question concerning whether they thought that a course in interprofessional learning would have any effect on their future relationships with other professional groups, almost 80 % answered ‘yes’. According to the participants, interprofessional learning should be included in undergraduate courses (90 %). Almost 80 % felt a greater understanding about problem-solving in teams within healthcare. Finally, 75 % of the participants thought a course in interprofessional learning would enable them to work more effectively as a member of a healthcare team.

On top of the annual evaluations of the students, teachers’, departments’ and faculties feedback and evaluations were also taken into account to adjust the program. Despite the fact that no structural gathering of this data was used, it seemed that participating institutions also adjusted their curriculum in order to better prepare students for an interprofessional module. Content was refined yearly when necessary. The learning goals and learning processes were improved by using the most recent teaching methods. For example, in many participating educational programs self- and peer assessment was not used as an evaluation tool. After participating in the IPCIHC-module, this evaluation method was introduced in different curricula. In 2005 the curriculum of physiotherapy had no training in basic communication skills. After evaluation of the participation education program, it seemed there was a need for training in communication skills and so from 2006 onwards it was introduced in their curriculum. So the entry level of students changed over the years between 2005 and 2014. Another example is that, at the time, the social workers, medicine and nursing program participants had never worked with ICF as a framework to facilitate the process for delivering a care plan. ICF today is introduced as a theoretical framework in different curricula.

## Discussion

The aim of this study was to evaluate education on ‘collaborative skills’ to be learned in an interprofessional module. The results suggest a success of this Belgian IPCIHC module for the participating students and institutions. But the continuous changes in healthcare and curricula still challenge the interprofessional committee of the IPCIHC-module to keep on ‘proving’ the advantages of learning with, from and about each other during undergraduate programs in healthcare.

The importance of interprofessional education and the link with competence seem clear from the literature [29–31]. Moreover stakeholders from the micro and meso level of chronic care organisation in Belgium, identified the lack of integration of care as one of the biggest weaknesses of today’s healthcare system, along with the unclear definitions of the roles and functions of health professionals involved in care processes [32]. Collaboration cannot just be ‘brought’ to practice by giving theoretical frames [33]. Interprofessional education modules should develop the competence of ‘collaborator in healthcare’ in an interprofessional team. Education and practice have to be linked through ‘learning while collaborating interprofessionally’ and vice versa [33]. Therefore it is difficult to translate all these needs and criteria from literature and practice in an education module for a specific context. But the hidden drive to keep going and to keep looking for qualitative and relevant education is the quality and safety of patientcare [34, 35].

Based upon the results we suggest students have to work with their own cases from internship and not with fictitious cases. Moreover, the module on interprofessional collaboration should be organised during or after internship. Yearly, in the development of the IPCIHC-module, the organising team used the results of the evaluations, the written feedback and the oral feedback of the students and tutors to modify the IPCIHC-module where necessary. Also, each year the participating institutions evolved. Participating in the IPCIHC-module enhanced the awareness of the importance of communication skills and had an impact on different participation curricula. Due to this impact the entry-level competence changed for most participating students between 2005 and 2014. Curricula became more interprofessional-oriented. Taking this into account and based on the participant evaluation results, the value of the face-to-face interprofessional education program has been respected despite the continuous changes on different levels. It appears from the participant evaluation results that participants’ knowledge on the roles and duties of the other healthcare professionals increased. This underbuilds the selected and executed teaching methods. In every session the learning goals were appointed and processed. It may well be that this explicit interprofessional learning module helped participants to be aware of the changes in their understanding of how other healthcare professionals work. But also it helped curricula developers to be aware of international changes of how to prepare students to think and work more interprofessionally. Even though participants claimed that one week is not enough to get acquainted with all professions and aspects from the results, it seems that almost 80 % replied that the IPCIHC-module will impact their future relationship with other professional groups. The majority of the participants suggested that the IPCIHC-module increased their understanding of problem-solving in teams and that the course will enable them to work more effectively as a member of a healthcare team. This perception could be the result of the reflective methods used, but should be further investigated in future research for its real effects. It is not only about looking for ‘interprofessional‘answers for the questions in the modules’ assignments, it is also about working in an interprofessional team with respect to the definition of learning with, from and about each other. Participants became familiar with the definition of interprofessional collaboration as described in the literature, but this also applied to the curricula developers. The students worked with cases from their own internship, for which they made an interprofessional care plan. During the week they learned to discuss ethical problems while taking ‘ethical’ decisions. Moreover, students developed their insights in the competence of interprofessional collaborators in healthcare. The curricula developers on the other hand became aware of competence needed for basic training, thus preparing their students to enter this interprofessional module. All participants worked with the same competence model to reflect and to assess the group functioning as well as the individual development on the core competences per role, as well as the functioning of their group. Still, one has to be aware that the IPCIHC-module only offers handles and triggers to develop the competence of an interprofessional collaborator and to develop more interprofessional curricula. Whereas context is an important influencer on interprofessional collaboration, research is needed to investigate the real effectiveness of undergraduate programs for patientcare in clinical practice settings. It seems a never-ending story in an always dynamic and interactive complex practice.

## Conclusion

We gathered data through a questionnaire and gave an overview of the content and development of the interprofessional educational module. Despite the fact it is only a participant evaluation, based on seven closed questions, it gives a descriptive overview of positive participants’ evaluation of the interprofessional module over 10 years. The challenge still remains to keep on educating future healthcare professionals to collaborate interprofessionally. More research on teaching methods and curricula development is needed to investigate the real effect of undergraduate programs for patientcare in practice.

## Abbreviations

IPCIHC: Interprofessional Collaboration In Healthcare
CanMeds: Canadian Medical Education Directions for Specialist
